# Evolutionary dynamics and functional characterization of proximal duplicated sorbitol-6-phosphate dehydrogenase genes in Rosaceae

**DOI:** 10.3389/fpls.2024.1480519

**Published:** 2024-11-08

**Authors:** Fan Yang, Jiawei Luo, Songxue Han, Yirong Zhang, Zhiguang Liu, Jincheng Lan, Yaqiang Sun, Tao Zhao

**Affiliations:** State Key Laboratory for Crop Stress Resistance and High-Efficiency Production/Shaanxi Key Laboratory of Apple, College of Horticulture, Northwest A&F University, Yangling, Shaanxi, China

**Keywords:** sorbitol, Rosaceae, S6PDH, proximal duplicated genes, synteny analysis, function divergence, gene transposition

## Abstract

Sorbitol is a critical photosynthate and storage substance in the Rosaceae family. Sorbitol 6-phosphate dehydrogenase (S6PDH) functions as the pivotal rate-limiting enzyme in sorbitol synthesis. The origin and functional diversification of S6PDH in Rosaceae remain unclear, largely due to the complicated interplay of gene duplications. Here, we investigated the synteny relationships among all identified S6PDH genes in representative genomes within the Rosaceae family. By integrating phylogenetic analyses, we elucidated the lineage-specific expansion and syntenic conservation of S6PDH across diverse Rosaceae plant lineages. We found that S6PDH can be traced back to a pair of proximal duplicated genes of the common ancestor of the Rosaceae, and the further amplification of S6PDH in the Maleae primarily relies on WGD events in their lineages. In Rosaceae species, multiple copies of the S6PDH gene are preliminarily divided into two main clades (Clade 1 and Clade 2) based on sequence similarity. These clades have evolved to acquire different functional directions. In Clade 1, lineage-specific transposition events in the Amygdaloideae have led to changes in gene expression patterns and promoted lineage evolution. This is mainly characterized by a decrease in enzymatic activity and transcriptional expression in the leaves, but also includes specific functional diversification, such as sustained post-harvest fruit expression and enhanced expression under biotic stress in certain tissues. In contrast, S6PDH in the Rosoideae and Dryadoideae has not undergone additional duplications beyond early proximal duplication. The loss of exons and variations in exon length might the key factor leading to reduced enzymatic activity in the Clade 2 proximal gene pairs. Collectively, our findings illuminate the dynamic nature of S6PDH evolution and reveal the intricate interplay between duplication, transposition, and functional diversification. This work not only contributes valuable insights into the genetic mechanisms underlying sorbitol metabolism but also establish a crucial foundation for future investigations aimed at comprehensively characterizing the variations of sorbitol metabolism across different subfamilies within the Rosaceae family.

## Introduction

The Rosaceae, recognized for its ancient origins and significant economic significance, comprises three subfamilies, sixteen tribes, and approximately three thousand species. Unlike most plants that use sucrose as their major photosynthetic end product and translocated carbohydrate, species within the Rosaceae, notably those in the Amygdaloideae subfamily, mainly utilize sorbitol as the form of transport for photosynthate and as a soluble storage substance (Zhou et al., 2006). Sorbitol 6-phosphate dehydrogenase (S6PDH, EC 1.1.1.200, also known as aldose-6-phosphate reductase) functions as the pivotal enzyme in sorbitol synthesis. It catalyzes the conversion of glucose-6-phosphate to sorbitol-6-phosphate, which is subsequently dephosphorylated to form sorbitol (Zhou et al., 2003). The interconversion between glucose-6-phosphate and sorbitol-6-phosphate is a reversible reaction, with S6PDH predominantly catalyzing the synthesis of S6P within Rosaceae species (Pleyerová et al., 2022; Hirai, 1979).

S6PDH activity has been identified in a variety of Rosaceae species (Hirai, 1979). By isolating the S6PDH protein from the leaves of *Eriobotrya japonica* and conducting enzymatic assays, researchers have determined that the most favorable pH for the oxidation of sorbitol-6-phosphate by S6PDH is precisely 9.8, at which the enzyme displays high activity within the pH range of 9 to 10. The optimal pH range for glucose-6-phosphate reduction is broader, with activity observed over a wider range of 7-9, compared to the narrower range for sorbitol-6-phosphate oxidation (Hirai, 1981). The S6PDH was purified from apple leaves, and the pH optimum for glucose 6-phosphate reduction was determined to be 9.5 (Negm and Loescher, 1981). Furthermore, the maximum velocity of G6P reduction by S6PDH was significantly higher than that of S6P oxidation, indicating a significantly higher catalytic capacity of S6PDH towards glucose-6-phosphate in apple (Kanayama and Yamaki, 1993). The leaves serve as the principal locale for the manifestation of S6PDH enzymatic activity within the majority of species belonging to the Rosaceae, while the enzymatic activity of S6PDH in fruits is typically low (Bortiri et al., 2002). At the subcellular level, S6PDH is mainly bound to thylakoid membranes and is also cytosol localized (Yamaki, 1981). The expression profile of genes encoding S6PDH exhibits a similar pattern to that of its enzyme activity (Sakanishi et al., 1998; Yamaki, 1981).

S6PDH gene expression was elevated in apples and peaches under stress (Kanayama et al., 2006). As S6PDH plays a pivotal role in sorbitol synthesis, many researchers have conducted transgenic studies to explore how changes in metabolic regulation during stress impact plant survival and competitiveness. The transformation of apple S6PDH gene into tobacco or persimmon resulted in enhanced plant stress resistance. However, the artificial accumulation of sorbitol in plants that naturally lack sorbitol, up to a threshold of less than 20 μmol/g FW, is associated with normal plant growth. Excessive sorbitol accumulation beyond this threshold can lead to growth disorders. In contrast, Rosaceae species, which utilize sorbitol as their primary carbon source, can tolerate remarkably high concentrations in their leaves without adverse effects on growth. For instance, peach leaves commonly exhibit sorbitol levels exceeding 100 μmol/g FW (Nadwodnik and Lohaus, 2008). Studies involving the sense and antisense transformation of S6PDH in apple have highlighted the enzyme’s crucial role in modulating the sorbitol-to-sucrose ratio in apple leaves (Kanamaru et al., 2004).

Gene duplication, a major driver of species evolution and gene function diversification, is a central focus in phylogenetic genomics (Qiao et al., 2019). Gene duplication events can be classified into several types, including tandem duplication, proximal duplication, decentralized duplication, and translocation duplication, as well as whole genome duplication (WGD) (Reams and Roth, 2015). A study has identified that there were three S6PDH genes in pear, revealing that single-gene segment duplication is the primary mechanism driving the amplification of S6PDH in pears (Qiao et al., 2018). Similar expansion of the S6PDH gene family has also been observed in apples (Li et al., 2022).

Synteny, which refers to the preservation of gene order on chromosomes throughout evolution, has proven to be an insightful approach for understanding the evolutionary connections among genes within and across species (Schultz et al., 2023). Synteny likely reflects important relationships between the genomic context of genes both in terms of both function and regulation, and thus is often used as a “proxy for the conservation or constraint of gene function” (Yang et al., 2023; Zhao et al., 2017; Zhang et al., 2020). Besides this conservation, the dispersion or fragmentation of gene clusters on a local scale is particularly noteworthy, as it has been demonstrated to induce alterations in regulatory expression by exposing key regulators to novel environments, thereby contributing to divergent gene expression and the emergence of morphological novelties (Liu et al., 2020; Lin et al., 2016). Utilizing network clustering to study syntenic relationships between genes presents a promising approach for investigating the synteny of gene families across different species, as it overcomes challenges imposed by pairwise interspecies comparisons and facilitates the identification of lineage-specific transposition quickly and accurately. This method allows for inferring the functional and new evolutionary trends of gene families (Zhao et al., 2017; Zhang et al., 2020). This approach not only accelerates the discovery of such transposition but also allows for a nuanced interpretation of their impact on the functional and evolutionary trajectories of gene families (Yang et al., 2023; Kerstens et al., 2020; Hammoudi et al., 2016).

It can be seen that many advances have been made in the cloning, identification and catalytic characteristics of S6PDH in a single species, and the involvement of sorbitol in abiotic stress regulation. Yet, when considering the Rosaceae family as an entirety, the research dedicated to unraveling the systematic evolution of the S6PDH gene remains strikingly deficient. This highlights the need for more extensive studies to gain deeper insights into the evolutionary history and functional implications of S6PDH across this diverse plant family. By combining phylogenetic reconstruction and synteny analysis, this study traced the ancestral origin of S6PDH in the Rosaceae lineage, followed by investigating the lineage-specific expansion and syntenic conservation in plant lineages of different Rosaceae subfamilies. This study unveiled previously undetected transposition of S6PDH and its regulatory expression responses in Amygdaloideae, thereby providing novel insights into the differences in sorbitol metabolism among different subfamilies of Rosaceae species.

## Materials and methods

### Plant genomes collection

In this study, we collected plant genomes from comprehensive databases. The selection encompassed representative taxa within the Rosaceae family including Amygdaloideae (*Malus domestica-cv.HanFu*, *Malus domestica-cv.Gala*, *Malus domestica-cv.GoldenDelicious*, *Malus sieversii*, *Malus sylvestris*, *Malus baccata*, *Pyrus x bretschneideri*, *Eriobotrya japonica*, *Gillenia trifoliata*, *Prunus persica*, *Prunus mume*, *Prunus armeniaca*, *Prunus avium*), Rosoideae (*Fragaria vesca*, *Rosa chinensis*) and Dryadoideae (*Dryas drummondii*). In addition to these, Arabidopsis (*Arabidopsis thaliana*), snapdragon (*Antirrhinum majus L*), grape (*Vitis vinifera*), and rice (*Oryza sativa*) were included as outgroups for comparative analysis. Detailed information regarding the genomes utilized in this study can be found in **Supplementary Table 1**. Each genome underwent a standardized naming and preprocessing step, and only the longest transcript was retained along with the corresponding BED/GFF annotation files for subsequent analyses.

### Identification of S6PDH family members in Rosaceae plants

The conserved domain (Aldo_ket_red: PF00248) of AKR, which the superfamily to which S6PDH belongs, was obtained from the Pfam database (http://pfam.xfam.org/) and the Hidden Markov model (HMM) was constructed. Using HMMER3 (Finn et al., 2011), an exhaustive search was conducted to extract all AKR sequences with an E-value below 0.001 from the genomes of twenty representative species. Sequences were subsequently annotated, categorized, and extracted by referencing the S6PDH sequences (**Supplementary Table 2**) which were previously reported.

### Sequence alignment and phylogenetic analysis

The S6PDH phylogenetic tree was constructed using various methods. Through various combinations of alignment software such as MAFFT (L-INS-I strategy) (Katoh and Standley, 2013), Muscle5 (Edgar, 2022), and *hmmalign*, sequence trimming with TrimAl (Capella-Gutiérrez et al., 2009) or manual methods, and tree-building tools like IQ-TREE (Nguyen et al., 2015) or FastTree (Price et al., 2009), different models were selected and optimization parameters were adjusted for phylogenetic tree construction. Comparative analysis is then performed on the results from these multiple approaches. The final phylogenetic tree was constructed using the method of combination of *hmmalign* for alignment, manual sequence trimming, PAL2NAL (Suyama et al., 2006) for sequence conversion, and IQ-TREE v2.1.2 (with the best-fit model TIM3+F+G4). This resulted in a phylogenetic tree with high supported and conforms to the evolutionary relationships among Rosaceae species. The visualized and annotation of the phylogenetic tree were performed using iTOL (http://itol.embl.de).

### Syntenic block detection and synteny network construction

The computation of synteny blocks and the construction of the synteny network were carried out using the SynNet-Pipeline synteny analysis developed by Zhao and Schranz (https://github.com/zhaotao1987/SynNet-Pipeline). Species protein sequence similarity searches were conducted using DIAMOND v0.9.1 (Buchfink et al., 2021). MCScanX (Wang et al., 2012) with default parameters was used to detect all possible syntenic associations between and within the genomes of different species. Syntenic blocks related to the S6PDH gene were extracted from the comprehensive synteny database to construct the target synteny network. The synteny relationships were annotated on the phylogenetic tree using iTOL, and the constructed network was visualized and annotated using Gephi v0.9.1 (Bastian et al., 2009).

### Gene expression profiles analysis

A total of 324 RNA-seq samples were used in this study, comprising 315 samples obtained from the SRA database and 9 samples generated through our experimental procedures. The RNA data of eight Rosaceae species (apple, pear, loquat, apricot, peach, plum, rose, and strawberry) were collected across various tissues, developmental stages, postharvest storage periods, and stress treatments. Initially, the raw data underwent filtering using Fastp v0.20.1 (Chen et al., 2018) to remove low-quality reads, adapter remnants, and other confounding factors. Subsequently, Kallisto v.0.46.1 (Bray et al., 2016) was used to build an index based on coding sequence (CDS) files. Clean reads from each sample were then mapped to their respective indexes to compute transcript TPM (Transcripts Per Kilobase of exon model per Million mapped reads) values. Detailed information about the RNA-seq samples can be found in **Supplementary Table 3.**

### Protein expression and purification

A total of eleven S6PDH sequences from Rosaceae species were selected for use. Two S6PDH sequences from apple were successfully cloned using the primers provided in **Supplementary Table 4**. The remaining nine S6PDH sequences from different species were synthesized by Sangon Biotech, and the corresponding sequence information is presented in **Table 1**. The full-length coding sequences of S6PDH obtained were respectively constructed into the pGEX4T-1 and PET-32a vectors (the optimal applicable vector for each sequence was determined based on the results of induction and purification). The recombinant plasmids were then transformed into the prokaryotic expression strain BL21 (DE3).

**Table 1:** Detailed information on the selected genes.

ID	Species	Subfamily	Clade	Class
*Mgde07G1054400*	*Malus domestica*	Amygdaloideae	Clade 1	Class A
*Mgde10G1062300*	*Malus domestica*	Amygdaloideae	Clade 2	Class B
*pbr_024722.1*	*Pyrus x bretschneideri*	Amygdaloideae	Clade 1	Class A
*pbr_042781.1*	*Pyrus x bretschneideri*	Amygdaloideae	Clade 2	Class B
*ppe_02G061100*	*Prunus persica*	Amygdaloideae	Clade 1	Class A
*ppe_08G083400*	*Prunus persica*	Amygdaloideae	Clade 2	Class B
*RochChr6G00021990*	*Rosa chinensis*	Rosoideae	Clade 1	Class B
*RochChr6G00021960*	*Rosa chinensis*	Rosoideae	Clade 2	Class B
*FvesH4_2g10630*	*Fragaria vesca*	Rosoideae	Clade 1	Class B
*Drydscaffold178G00000100*	*Dryas drummondii*	Dryadoideae	Clade 1	Class B
*Drydscaffold178G00000130*	*Dryas drummondii*	Dryadoideae	Clade 2	Class B

Single colonies transformed with the recombinant plasmids pET-32a/*Mgde07G1054400*, pET-32a/*Mgde10G1062300*, pET-32a/*pbr_024722.1*, pGEX4T-1/*pbr_042781.1*, pET-32a/*Drydscaffold178G00000100*, pET-32a/*Drydscaffold178G00000130*, pGEX4T-1/*RochChr6G00021990*, pET-32a/*RochChr6G00021960*, pGEX4T-1/*ppe_02G061100*, pET-32a/*ppe_08G083400*, and pET-32a/*FvesH4_2g10630* were picked from LB plates and inoculated into 5 mL of LB liquid medium (containing 100 mg/L Amp). They were cultured overnight at 37°C. Overnight cultures were diluted 1:100 into 100 mL of LB medium containing Amp, and incubated at 37°C, 250 rpm for 3.5 hours (until OD600 reached approximately 0.6). A 1 mL culture served as an uninduced control for electrophoresis. IPTG was added to the remaining culture to a final concentration of 0.3 mM, and induction was carried out at 16°C to 37°C (optimal induction temperature determined according needed for each protein) for 16 to 20 hours. The induced bacterial suspension was centrifuged (4°C, 12000g, 5 min), the supernatant discarded, and the sediment resuspended in 1× PBS buffer. After centrifugation (4°C, 12000g, 5 min), the supernatant was removed, and the bacterial pellet was resuspended in 1× Ni-NTA binding buffer with lysozyme and protease inhibitors added to a final concentration of 1 mg/mL. Under an ice bath, the *E. coli* was disrupted through sonication and then centrifuged at 4°C for 15 min. The protein-containing supernatant was collected. The supernatant was passed through a His or GST affinity chromatography column at low temperatures, the eluted protein was collected in 1.5 mL centrifuge tubes sequentially, multiple tubes of highly purified soluble protein were obtained. From each tube, 20 μL of the eluted protein was combined with 5× loading buffer. Similarly, the reserved bacterial controls were also mixed with 5× loading buffer. Both samples were subjected to boiling for 10 minutes prior to being analyzed via SDS-PAGE gel electrophoresis. Proteins with high content and purity proteins were chosen for subsequent protein quantitation and enzyme activity assays.

### Protein content and enzyme activity assay

Protein content was measured by the BCA protein assay kit. Each experiment was independently repeated four times. The specific operation procedures were referred to the instructions attached to the kit. A standard curve was plotted to calculate the regression equation as follows: y = 0.188x - 0.0046, *r* = 0.9987 (the standard curve is shown in **Supplementary Figure 1**).

Enzyme activity for recombinant purified S6PDH protein was assessed using Sorbitol-6-Phosphate Dehydrogenase assay kit. The measurement is based on the reduction of G6P catalyzed by S6PDH, resulting in NADPH oxidation. By quantifying the rate of decline in NADPH absorbance at 340 nm, the activity of the purified S6PDH enzyme can be calculated.

## Results

### Phylogenetic reconstruction of AKR and distribution of S6PDH in Rosaceae

S6PDH is a member of the Aldo-Keto Reductases (AKRs), a family of enzymes characterized by the Aldo-Keto Reductase domain (PF00248). These enzymes catalyze the NAD(P)H-dependent reduction of various natural and exogenous carbonyl-containing substrates to primary and secondary alcohols. The AKR superfamily is known to encompass a versatile array of enzymes such as xylose reductase, galactose dehydrogenase, aldehyde reductase, and aldose-6-phosphate reductase (Mindnich and Penning, 2009; Penning et al., 2019). In this study, a total of 877 AKR proteins were identified from 20 plant genomes, representing diverse taxa within the Rosaceae family. The aforementioned sequences were utilized for the reconstruction of a phylogenetic tree of the AKR superfamily, to which S6PDH belongs. Through an examination of the phylogenetic tree’s topology, branch support values, as well as functional annotation of selected AKR sequences from Arabidopsis, along with reported functional AKR sequence data from other species, distinct groups within the AKR superfamily were delineated. The phylogenetic tree revealed the classification of AKRs into 16 classes, including members of the S6PDH (Sorbitol-6-phosphate dehydrogenase) family crucial for sorbitol metabolism in Rosaceae plants. Notably, the bootstrap values for all major branches exceeded 94%, indicating a high degree of confidence in the classification.

**Figure 1:**
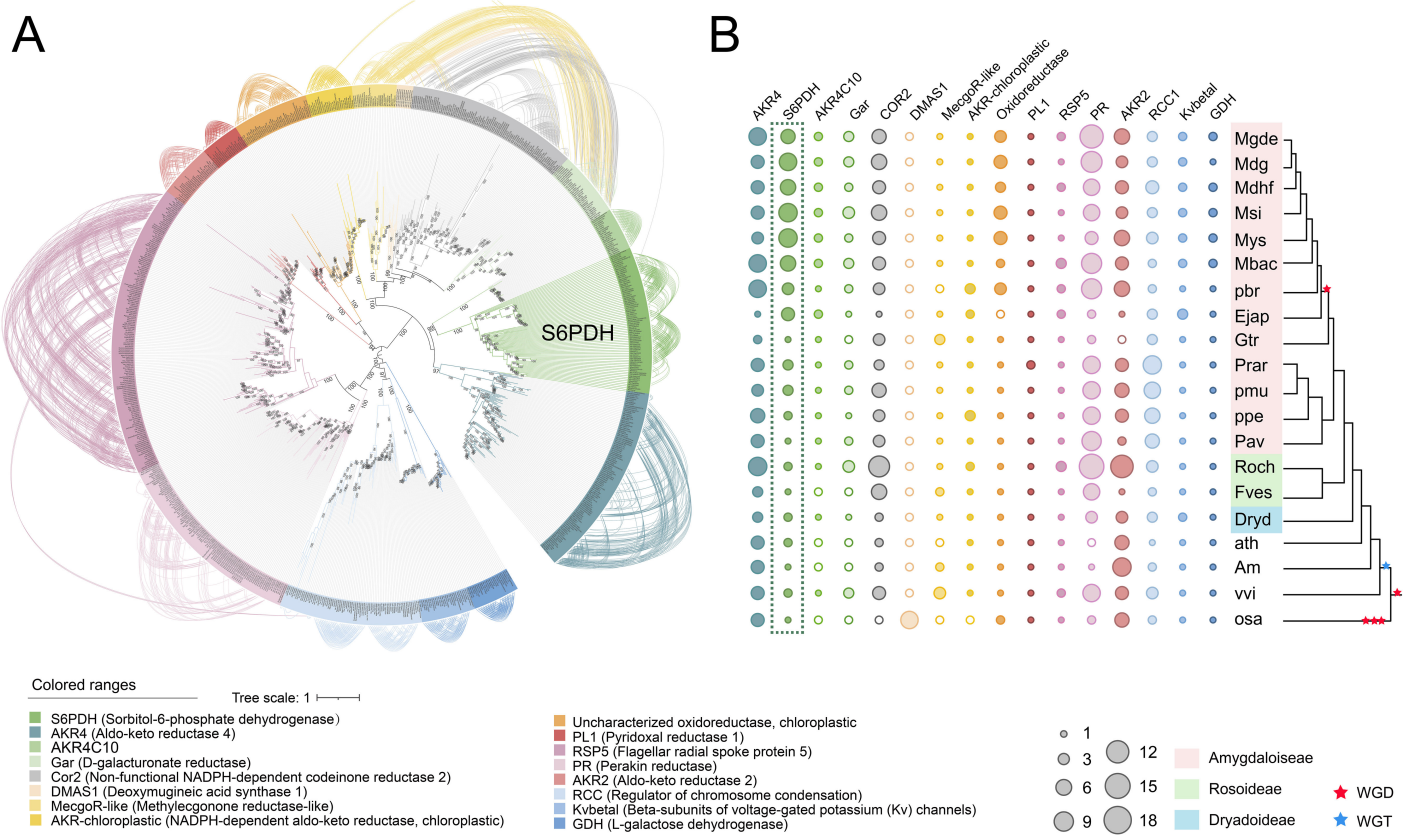
Phylogenetic tree and distribution of the AKR family in Rosaceae. **(A)** Maximum-likelihood tree for the AKT gene family and syntenic relationships between the genes. Terminal branch represent 16 categories, with the corresponding color and category names displayed beneath the Phylogenetic tree. Each connecting line located outside the circular gene tree indicate strong conservation of synteny between gene pairs within subclades. The connecting lines were colored according to the corresponding communities. **(B)** The copy numbers of various AKR categories for each plant species. The degree of enrichment was represented by the circle size. Species names were shown on the right side. Red and blue pentagrams on the tree indicate known whole-genome duplication (WGD) and whole-genome triplication (WGT) events, respectively. The corresponding phylogenetic trees of these species were provided. The AKR categories ID were indicated at the top of the figure. Species abbreviations were as follows: Mgde, *Malus domestica* cv.GoldenDelicious; Mdg, *Malus domestica* cv.Gala; Mdhf, *Malus domestica* cv.HanFu; Msi, *Malus sieversii*; Mys, *Malus sylvestris*; Mbac, *Malus baccata*; pbr, *Pyrus x bretschneideri*; Ejap, *Eriobotrya japonica*; Gtr, *Gillenia trifoliata*; ppe, *Prunus persica*; pmu, *Prunus mume*; Prar, *Prunus armeniaca* L; Pavi, *Prunus avium*; Fves, *Fragaria vesca*; Roch, *Rosa chinensis*; Dryd, *Dryas drummondii*; ath, *Arabidopsis thaliana*; vvi, *Vitis vinifera*; ama, *Antirrhinum majus* L; osa, *Oryza sativa*.

The construction of an undirected synteny network related to AKR was undertaken in order to further investigate the evolutionary relationship between S6PDH and other members of the AKR family (**Figure 1A**). The network encompasses 594 nodes (representing the number of AKRs located in the synteny regions), and 3,890 edges (representing the number of paired synteny connections between AKRs). When the network is mapped onto the phylogenetic tree, strong synteny signals are displayed within each branch (**Figure 1A**), further supporting the reliability and accuracy of the AKR family classification proposed earlier based on phylogenetic relationships.

The copy numbers within Rosaceae species exhibit obvious variation across AKR subfamilies, which aligns with the birth-death model of gene family evolution wherein genes generated through gene duplication can either be retained or lost over time (**Figure 1B**) (Panchy et al., 2016). The S6PDH gene was conserved across all examined species, with a higher number of copies observed in the subfamily Amygdaloideae, particularly within the Maleae species. The substantial retention of S6PDH copies in Amygdaloideae species is consistent with the generally high levels of sorbitol present within this subfamily, which reflects the pivotal role played by S6PDH in facilitating the metabolism and accumulation of sorbitol within Rosaceae.

In addition, there was a distinct variation in the distribution of S6PDH copy number between wild and cultivated apples. The wild species, exemplified by *Malus sieversii* and *Malus sylvestris*, each manifest 10 copies of the S6PDH gene. By contrast, cultivated varieties maintain a diminished number of S6PDH gene copies. It was found that cv. Golden Delicious has 6 copies, cv. Hanfu contains 7 copies, and cv. Gala retains 9 copies (including two S6PDH fragments). The copy number of the S6PDH gene in wild apple species was generally higher than that in cultivated cultivars, suggesting a divergent evolutionary history for S6PDH between wild and cultivated apple varieties. The number of S6PDH gene copies preserved in cultivated apples remains significantly higher compared to many other species (**Supplementary Figure 2**). This suggested that S6PDH holds an exceptionally significant role within the apple’s genetic system.

### Phylogenetic reconstruction of the S6PDH gene family in Rosaceae

To further investigate the expansion process and genetic characteristics of the S6PDH gene family within the Rosaceae lineage, including species from Amygdaloideae (n = 13), Rosoideae (n = 2), and Dryadoideae (n = 1), totaling 16 genomes), we screened S6PDH sequence from AKR protein sequence and reconstructed phylogenetic tree (**Supplementary Table 1**). However, the phylogenetic tree constructed based on protein sequences exhibited weak bootstrap support and its topological structure was unable to accurately reflect the relationships between species (**Supplementary Figure 3, Supplementary Figure 4**). These results indicate that the S6PDH protein sequences may contain insufficient informative sites, thereby posing a challenge to the construction of a highly precise phylogenetic tree. To resolve this issue, pal2nal was used to convert amino acids into corresponding nucleotides, which marked improved the number of available information sites and provides richer genetic data for constructing high-quality phylogenetic trees. In addition, we performed detailed manual sequence pruning to remove problematic codons, selected different models, and adjusted optimization parameters to construct phylogenetic trees. The S6PDH phylogenetic tree was successfully constructed, exhibiting a robust bootstrap support (branch Bootstrap ≥ 90%) and the obtained phylogenetic relationships among the S6PDH sequences align well with the established phylogenetic relationships of Rosaceae plant species, laying the foundation for the subsequent analysis and research.

**Figure 2:**
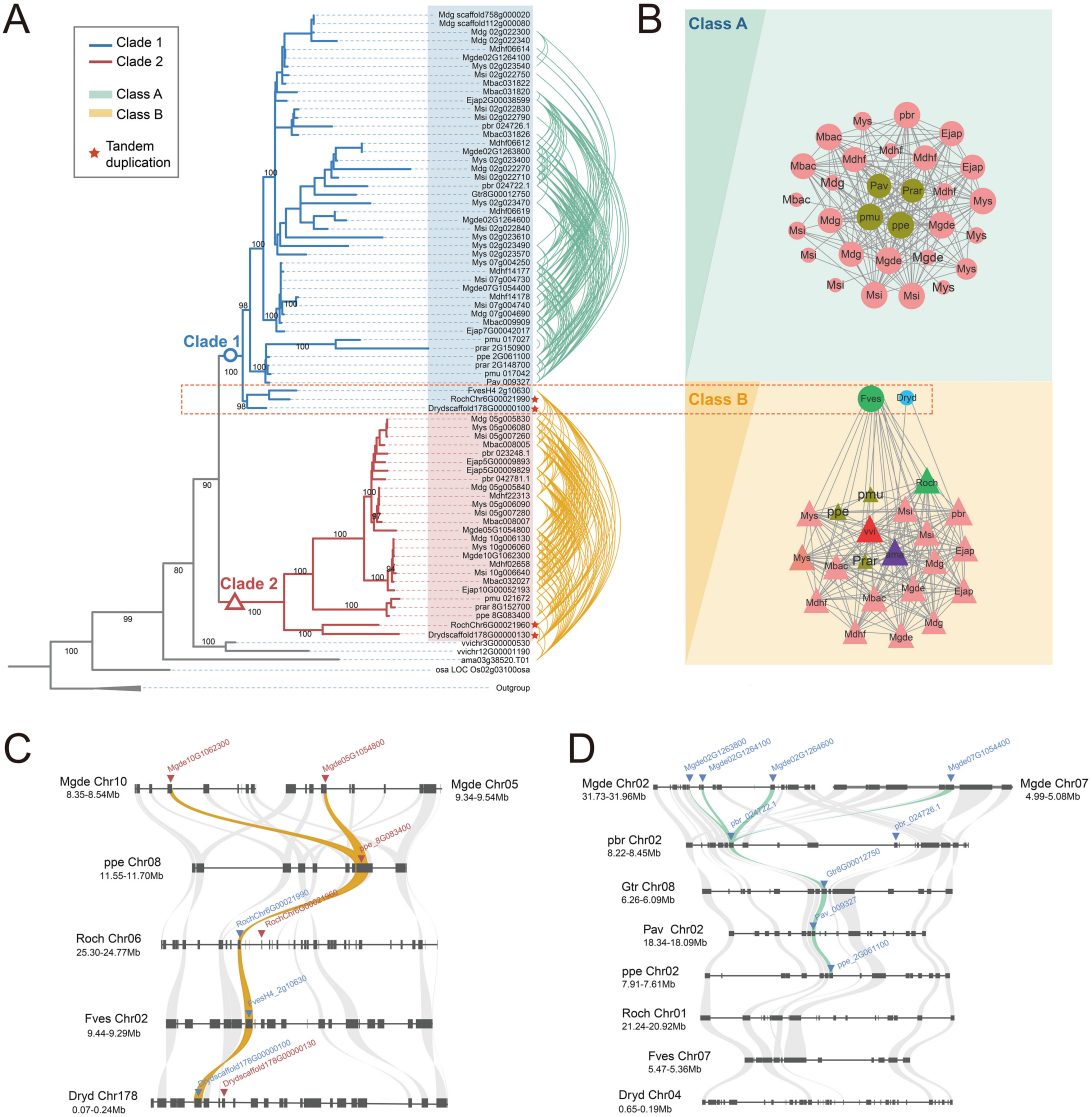
Phylogenetic reconstructions and synteny analysis of the S6PDH in Rosaceae. **(A)** The phylogenetic tree was constructed using S6PDH sequences from representative Rosaceae genomes and rooted with sequences attributed to several non-S6PDH genes within the AKR superfamily. Branch lengths represent divergence times, while bootstrap values were indicated for the main internal branches. The genes are colored blue or red based on their corresponding clade. Curves connect the identified syntenic genes. The connecting lines of green and orange are color-coded based on their corresponding communities, Class A and Class B, respectively. **(B)** The synteny network of S6PDH in Rosaceae species can be divided into conserved cluster (Cluster B) and Amygdaloideae lineage-specific cluster (Cluster A), where the background color corresponds to class. The size of each node corresponds to its node degree, representing the number of edges it has. The nodes were colored based on their taxa: Amygdaloideae (Maleae in pink, Prunus in brown), Rosoideae (green), and Dryadoideae (blue). The letters used in the nodes represent the species. The shape indicated the clade, where triangles correspond to clade 2 and circles correspond to clade 1. **(C, D)** Microsynteny relationships of homologous genomic segments of the S6PDH genes across various Rosaceae species. The S6PDH genes from Clade 2 were indicated by red arrows, while those from Clade 1 were indicated by blue arrows. The syntenic gene pairs were connected by curved lines, with the ones belonging to the Class B cluster highlighted in orange and those belonging to the Class A cluster highlighted in green.

According to the phylogenetic tree (**Figure 2A**), the S6PDH clustered into two major clades based on species composition and bootstrap support values (Bootstrap = 90%), designated as Clade 1 and Clade 2. Both clades encompass the S6PDH genes from all species used in Rosaceae. The cross-clade proximal duplication of S6PDH was observed in Rosoideae and Dryadoideae. For concreteness, the gene pairs, *Drydscaffold178G00000130* and *Drydscaffold178G00000100*, which belong to Clade 2 and Clade 1 respectively, exhibited a clear proximal sequential pattern in arrangement (**Supplementary Figure 5** and **Supplementary Table 5**). The same proximal duplcation pair was detected in rose (*RochChr6G00021960* and *RochChr6G00021990*). The observation suggested that the S6PDH in Rosaceae could be traced back to a proximal duplication event in the common ancestor of Rosaceae. There were differences in the distribution of S6PDH between species of Prunus tribe and Maleae tribe. The S6PDH of Prunus tribe species was distributed in both Clade 1 and Clade 2, exhibiting no increased or decreased in duplication compared to Rosoideae and Dryadoideae species (**Figure 2A**). However, the S6PDH of Prunus tribe species deviated from the proximal arrangement pattern observed in Rosoideae and Dryadoideae species, instead being located on two distinct chromosomes (**Figures 2C, D**). The Maleae species demonstrated a higher degree of S6PDH multiplicity and possessed a greater number of copies of the S6PDH gene, which were distributed across four distinct chromosomes.

### Lineage-specific gene duplications and transpositions of S6PDH in Amygdaloideae

The synteny information of genomes, which refers to the similarity in gene arrangement on chromosomes across different genomes, can reflect the common origin of genomes and compare the rules of gene arrangement at a structural level (conserved or specific) (Zhao et al., 2017, Zhao et al., 2021). We obtained the S6PDH gene cluster using the synteny network process developed in the laboratory, and analyzed the gene arrangement rule and species distribution within the cluster to extract the synteny characteristics of S6PDH in the genomes of Rosaceae species. By combining phylogenetic tree reconstruction and synteny network analysis, we traced the evolutionary process of two branches of Rosaceae S6PDH and investigated the internal relationship between sequence duplication and subsequent divergence.

Two major gene clusters, namely Class A and Class B (**Figure 2B**), were identified through clustering (**Figure 2B**). By mapping the synteny relationships onto the phylogenetic tree (**Figure 2A**), it can be observed that not all S6PDH sequences present in the phylogenetic tree were reflected in the synteny network. For example, the proximal duplications of rose and Dryas retained only one representative node each in the synteny network. This discrepancy might arise due to certain genes being arranged in proximal, indicating their close proximity and resulting in a representative member of the gene cluster within the synteny network to avoid redundancy. Within the same species of Maleae, two nodes (representing copies of the S6PDH gene) derived from different chromosomes fell into the same synteny cluster and are interconnected. The presence of this pattern confirms that the duplication of S6PDH, resulting from the WGD event specific to the Maleae, occurred in both the Clade1 and Clade2 branches. The conservation of synteny across different chromosomes provided compelling evidence for the influence of the ancestral WGD event on the distribution of S6PDH genes in Maleae species (**Supplementary Figure 6**).

Noteworthy, the S6PDH sequences formed Clade 2, together with the selected outgroup species sequences, as well as the sequences from Rosoideae (*FvesH4_2g10630*, *RochChr6G00021990*) and Dryadoideae (*Drydscaffold178G00000100*), collectively constituted the cluster B. This cluster showed the conserved synteny among these sequences, which spanning multiple subfamilies within the Rosaceae family and incorporating the outgroup species. Despite sharing a high level of sequence similarity with the sequences from the Rosoideae and Dryadoideae subfamilies within Clade 1 in the phylogenetic tree, the S6PDH sequences from Amygdaloideae demonstrate altered chromosomal positioning information, leading to the formation of a separate lineage-specific synteny gene cluster known as Class A.

The results obtained from the microsynteny analysis offer supplementary evidence to support the aforementioned observation. The S6PDH sequences from Clade 2, as well as those from Rosoideae and Dryadoideae within Clade 1, exhibited a robust synteny relationship (**Figure 2C**). The S6PDH gene within the Amygdaloideae of Clade 1 showed a robust symbiotic relationship (**Figure 2D**). Despite the flanking gene retaining a high synteny relationship, the corresponding homologous sequence is missing in the synteny block of Rosoideae and Dryadoideae. The microsynteny pattern was consistent with the findings of the S6PDH specific synteny network cluster analysis in Amygdaloideae, which confirmed that the S6PDH sequences indeed underwent a lineage-specific transposition event in Amygdaloideae.

### Transposition of S6PDH in Amygdaloideae decreased S6PDH enzyme activity

Gene transpositions have been confirmed to alter gene expression patterns and drive lineage evolution. A case in point is the transposed MAF1 gene in Arabidopsis, where its transcript exhibits more abundant expression levels before vernalization compared to those of other MAR family members, and transposition promotes late flowering (Madrid et al., 2021). The transposition events involving the S6PDH gene within the Amygdaloideae lineage could play a crucial role in the diversification of S6PDH within Rosaceae, which have similarly initiated modifications in expression regulation, thereby triggering differences in functional retention of S6PDH. The aforementioned hypothesis was tested by selecting 11 representative sequences covering six species, including Amygdaloideae, Rosoideae, and Dryadoideae (**Table 1**).

**Figure 3:**
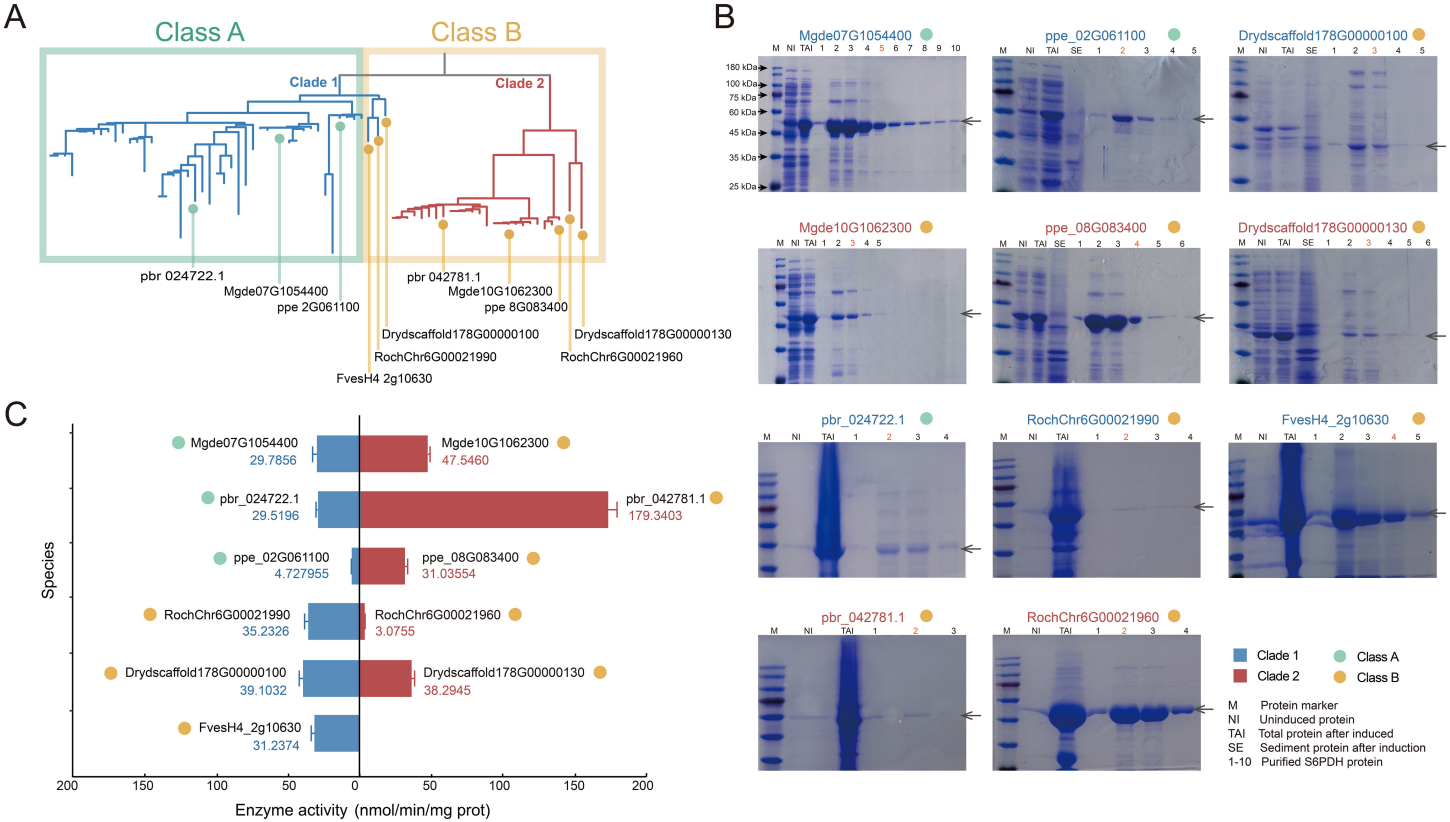
Expression of S6PDH recombinant protein. **(A)** The positioning of genes on the phylogenetic tree. Species abbreviations were as follows: Mgde, *Malus domestica*; pbr, *Pyrus x bretschneideri*; ppe, *Prunus persica*; Dryd, *Dryas drummondii*; Roch, *Rosa chinensis*; Fves, *Fragaria vesca*. **(B)** The SDS-PAGE gel electrophoresis results of the protein. The target protein bands were marked with arrows. The protein IDs were labeled above the gel image. Different color of IDs indicated the branches of the sequences on the phylogenetic tree, while the color of dot behind the IDs represented the synteny gene clusters in which the sequences were located. **(C)** S6PDH enzyme activity (n = 3 for each group). The bars depict the enzyme activity, with the mean values presented alongside their respective columns. The bar colors were assigned based on the gene branch in the phylogenetic tree, while the dot colors were determined according to the gene cluster affiliation.

The proteins were expressed and purified in the prokaryotic expression system. In order to ensure the comparability of enzyme activity among genes within the same species, simultaneous extraction and purification of S6PDH protein pairs were conducted. The SDS-PAGE analysis revealed expression bands for each S6PDH protein, with molecular weights consistent with the predicted fusion protein size, confirming successful expression of all 11 full-length S6PDH genes (**Figure 3B**). Subsequently, proteins with high protein content and purity were selected for further determination and comparison of enzyme activity, aiming to assess the differences in catalytic activity between pairs of S6PDH genes.

The gene pairs arranged in proximal from species of Rosoideae and Dryadoideae were located on two separate branches of the phylogenetic tree. (**Figure 3A**). Comparative analysis of S6PDH sequence enzymatic activities between rose and Dryas showed that, while the sequences in Clade 1 (*RochChr6G00021990* and *Drydscaffold178G00000100*) displayed higher enzymatic activity, it is notable that all sequences from these two species clustered within the conserved synteny cluster designated as Class B (**Figure 3C**). This phenomenon not only confirmed the conserved high enzyme activity characteristic of the sequences in the Class B cluster, but also implied that the high enzyme activity might arise from a shared genetic background and regulatory environment.

For the Amygdaloideae species, the superiority of enzyme activity was uniformly reflected in the conserved S6PDH sequence, which was anchored within Clade 2 and classified under the Class B synteny network (**Figure 3C**). Given the robust enzymatic activity exhibited by the sequences in Rosoideae and Dryadoideae within Clade 1 mentioned above, it is plausible that the common ancestor of this clade originally possessed a highly enzymatic phenotype. However, contrary to expectations, the sequences in Clade 1 that underwent a lineage-specific transposition event in Amygdaloideae did not augment enzyme activity but rather resulted in a reduction in enzyme activity. This suggested that the transposition event may have exerted an adverse effect on Amygdaloideae S6PDH protein activity instead of the anticipated enhancement.

Strawberry has only one S6PDH sequence located in Clade1, which is also classified as a synteny Class B cluster and exhibits high levels of enzyme activity *in vitro*. The obtained result contradicts the experimental findings of S. Duangsrisai et al., who observed the presence of S6PDH mRNA in strawberry leaves and fruits but did not detect its protein activity (Duangsrisai et al., 2007). The discrepancy in the outcomes of this enzyme activity detection may be attributed to the utilization of a mere 461 bp cDNA fragment for *FaS6PDH* in previous studies, whereas the present experiment employed the complete 930 bp strawberry S6PDH cDNA sequence. The variation in the length of the cDNA fragment can impact the structure and functionality of the protein, leading to disparities in enzyme activity detection outcomes. The utilization of full-length cDNA sequences for expression and enzyme activity detection is more likely to preserve the complete functionality of the protein, which may account for our successful detection of enzyme activity in our experiments.

### Sequence and structural differences of S6PDH in Rosaceae

**Figure 4:**
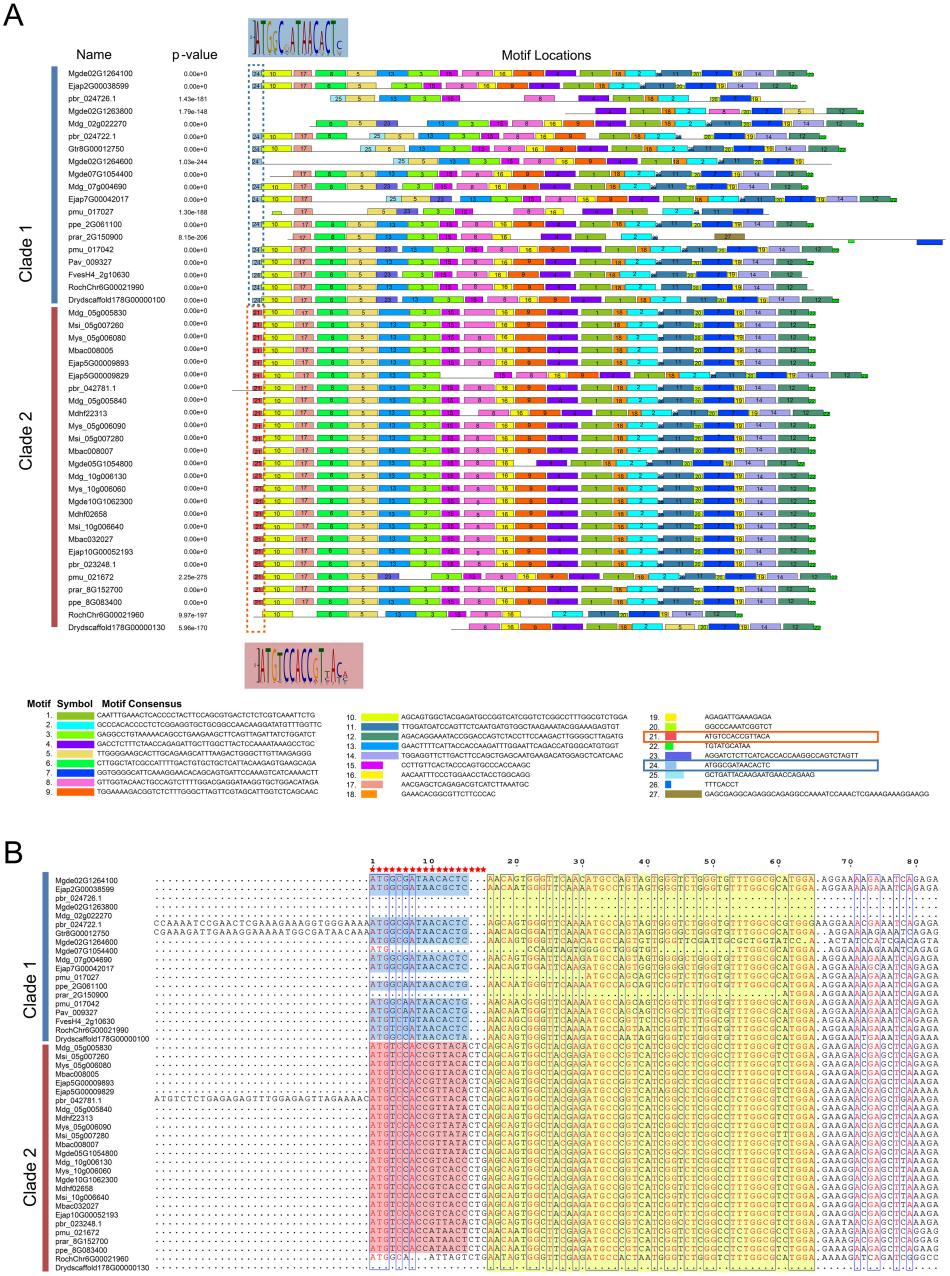
The prediction of key nucleotides sites conserved by S6PDH. **(A)** The characteristic sequence motif fingerprints of Clade 1 and Clade 2 in S6PDH are depicted, with each motif represented in a distinct color (ranging from Motif 1 to Motif 27). The conserved motifs were predicted using the MEME online server (http://meme-suite.org/tools/meme). Highlighted in the figure are the differential motifs (specifically, motifs 21 and 24) that distinguish Clade 1 from Clade 2 within S6PDH. The WebLogo was displayed, maintaining a consistent background color for clarity. **(B)** A partial sequence alignment of S6PDH is presented. The alignment was visualized using the ESPript 3.0 program (https://espript.ibcp.fr/ESPript/ESPript/index.php). The region corresponding to the framed motif in Figure A was highlighted, employing the same color scheme for consistency. The red pentagrams represent different key residues.

The comparative analysis of homologous S6PDH genes across the two evolutionary branches of Rosaceae suggested that Clade 2 appeared a higher degree of sequence consistency (**Figure 4**). Distinctive differences were noted in the N-terminal nucleotide motifs between sequences from Clade 2 and Clade 1 (**Figure 4**). For instance, the codons CTC/CTG (coding for leucine) were present at positions 16-18 in Clade 2, whereas they were conspicuously absent in Clade 1. This divergence in sequence initially prompted speculation regarding whether such discrepancies could be another potential cause of the reduced activity in the Clade 1 S6PDH homologues within the Amygdaloideae lineage, in addition to translocation. However, upon closer examination, this hypothesis was refuted: despite the N-terminal sequence variations, the experimental results indicated that the enzyme activity of sequences from Clade 1, particularly those from Dryas and rose, displayed high enzymatic activity. This finding negated the supposition that differences in the N-terminal sequence were responsible for diminished enzyme activity in the Clade 1. Consequently, it appeared that transposition events, rather than inherent sequence heterogeneities, were the primarily factor influencing the variation in enzymatic activity among the S6PDH duplication pairs in Amygdaloideae.

**Figure 5:**
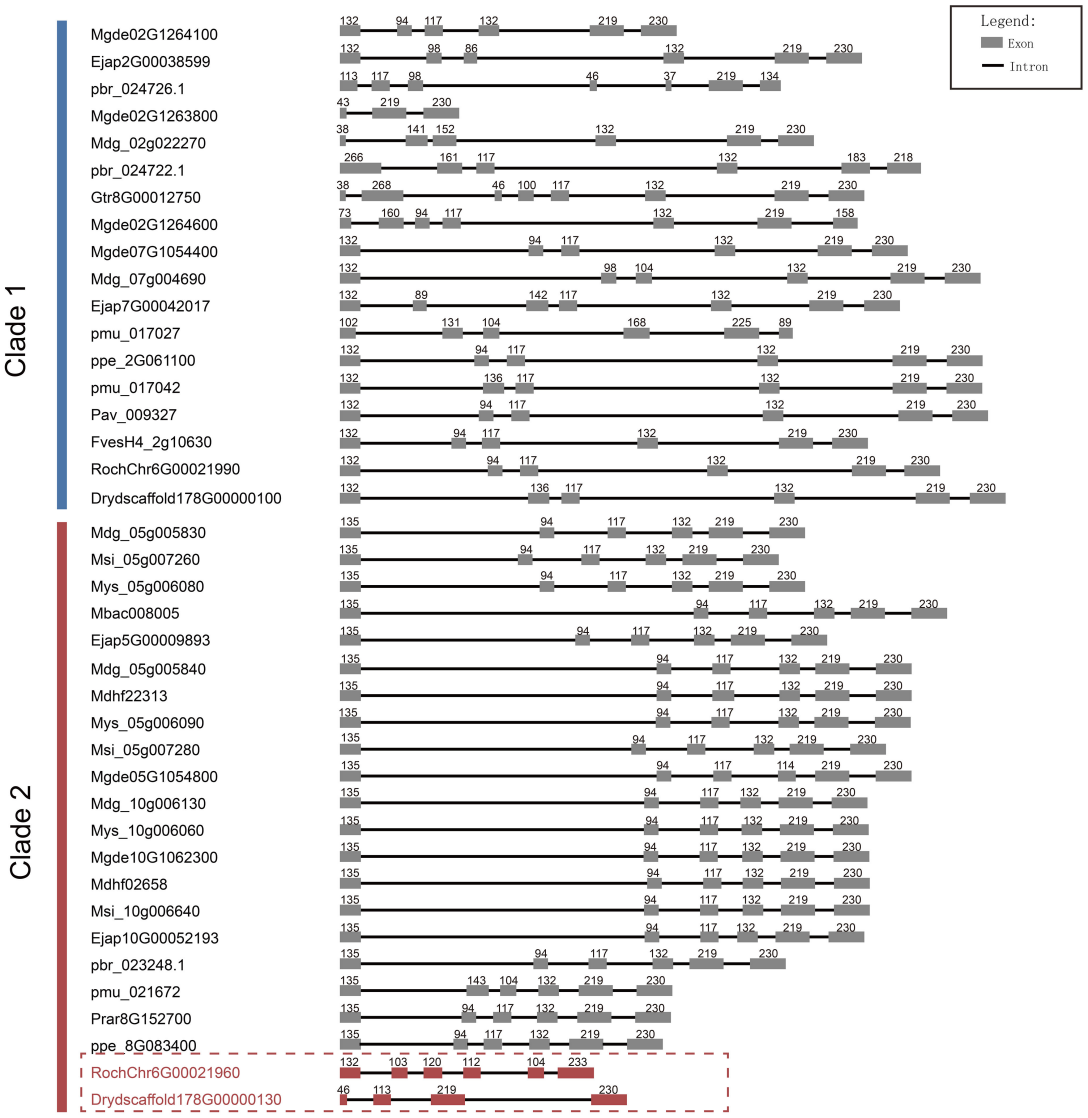
Length and number comparison of S6PDH multiple sequence exons/introns. Exons are depicted as boxes, and introns are represented by lines. The numbers above each box denote the nucleotides length of the exons. The sequence IDs were shown on the left.

Furthermore, through comparative analysis of proximal duplicated gene pairs from rosa and Dryas species, we observed that the sequences *RochChr6G00021960* and *Drydscaffold178G00000130* within Clade 2 exhibited N-terminal deletions relative to other homologous sequences in Rosaceae (**Figure 4**). In conjunction with structural analyses (**Figure 5**), it becomes evident that S6PDH sequences in Rosaceae typically encompass six exons, with a high degree of consistency in the length of each exon sequence across the family. Unlike the other sequences, the S6PDH from Dryas situated within Clade 2 (*Drydscaffold178G00000130*) had distinct exon deletion, retaining only four exons. Although the S6PDH of rosa located on Clade 2 (*RochChr6G00021960*) contains 6 exons, with varying exon lengths that deviate from the normative patterns observed in other sequences. Alterations in exon structure have been documented to impact the protein coding regions, which can cause in-frame deletions of functional protein domains or generate novel reading frames through frameshifts (Wang et al., 2021). The composition of the protein domain or the adjustment of the functional region was affected, leading to interference with the stability of the protein or conformation of the active center, ultimately resulting in indirect or direct impact on enzyme catalytic efficiency (Wang et al., 2021). The aforementioned findings suggest that the deletion and length variation of S6PDH exons in Dryas and rose may be the important factors contributing to the reduced enzyme activity of Clade 2 proteins in the proximal gene pairs. These architectural changes could have played a crucial role in shaping the evolutionary trajectory of the gene pair.

### Expression profiles reveal S6PDH of Rosaceae undergo functional diversification

To deepen our understanding of the retention and differentiation of expression patterns following proximal duplication of S6PDH in Rosaceae, we conducted an analysis of S6PDH from the perspective of transcriptional regulation. We integrated transcript data from various tissues of Roseaceae species, including representatives of the Amygdaloideae (apple, pear, loquat, plum, apricot, peach), as well as representatives of Rosoideae (rose and strawberry) (**Supplementary Table 3**).

**Figure 6:**
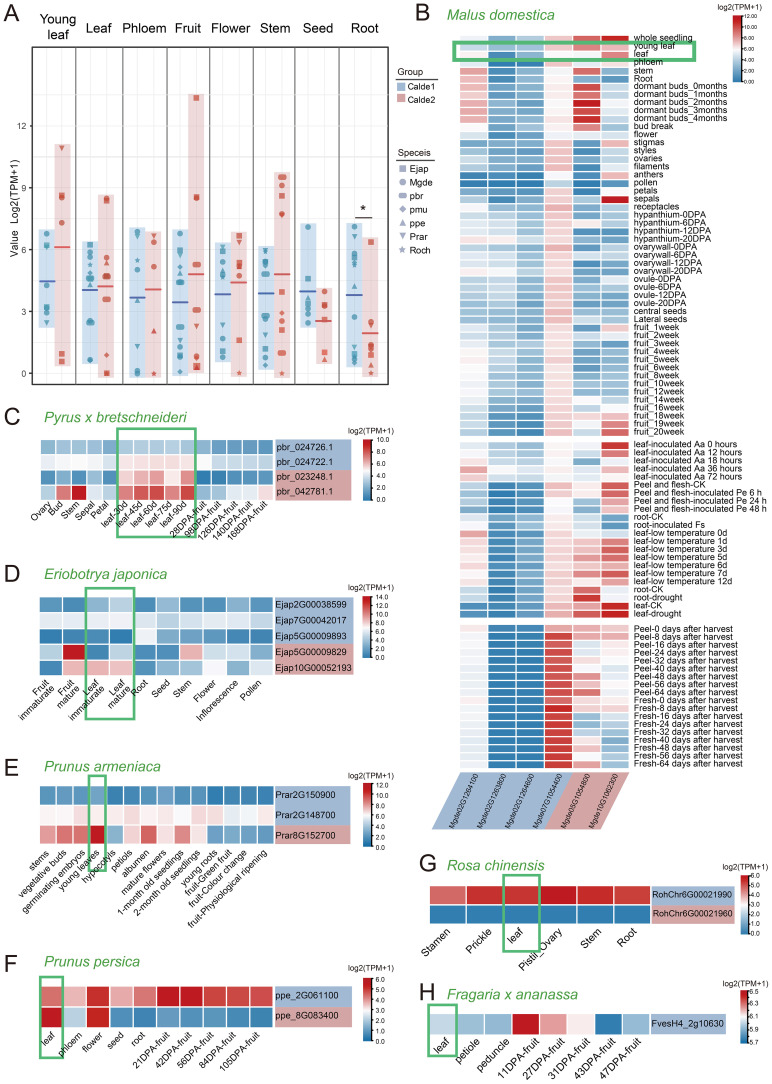
The transcriptional profiles of S6PDH in different Rosaceae species. **(A)** Plots of expression differences between gene pairs for Clade 1 and Clade 2 are shown, with the middle horizontal line indicating the average value. Different shapes indicate the species to which the sequence belongs. The background color corresponds to the clade to which the sequences belong. * indicates significant difference at the 0.05 levels by t-test. **(B-H)** Expression patterns of S6PDH in different tissues and throughout various developmental stages of Rosaceae species. The background color of each gene name is consistent with the clade to which the gene belongs. Abbreviations are as follows: Aa, *Alternaria alternata*; Pe, *Penicillium expansum*; Fs, *Fusarium solani*.

The S6PDH gene was broadly expressed across different organs, displaying both overlapping and distinct expression patterns in various tissues of Rosaceae species. Overall, the S6PDH genes in Clade 2 showed high average expression levels in leaves, fruits, flowers, and stems, while the S6PDH genes in Clade 1 exhibited a strong expression preference in seeds and roots, with particularly significant differences observed in the roots. (**Figure 6A**). Leaves are the main sites of sorbitol synthesis (Boris et al., 2017). The expression of S6PDH in leaves is essential as it serves as a pivotal enzyme in the sorbitol synthesis pathway. Attention was initially directed towards examining the expression patterns of S6PDH in the foliage of various Rosaceae species. The expression level of S6PDH Clade2 in leaves is generally higher than that of homologous copies of Clade1 in both Maleae (apple, pear, and loquat) and Prunus (peach, plum, and apricot) within the Amygdaloideae (**Figures 6B–H**). From a synteny perspective, these highly expressed sequences were conserved S6PDH without transposition. The S6PDH with lineage-specific transcripts from the Amygdaloideae had comparatively lower expression levels compared to homologous counterparts within the same species, indicating that transposition events exerted an inhibitory effect on gene transcription in leaves.

Interesting, transposition genes were found to evolved specific expression patterns. For instance, one transposed gene (Mgde07G1054400) in apple exhibited unique and strong transcriptional activity in the peel and flesh of fruit during storage (**Figure 6B**). Moreover, the transcriptional enhancement of the S6PDH genes, which have undergone transposition on chromosome 2 of apple, was observed in response to biological stress (**Figure 6B** and **Supplementary Figure 7**). In contrast, the expression of conserved S6PDH in Clade2 showed upregulation during late leaf and fruit development as well as under abiotic stress conditions. The transcriptional specificity is likely to be intricately linked to transposition, which, throughout evolution, has bestowed these genes with distinctive transcriptional functions that enable them to operate during specific developmental stages or under particular environmental conditions. It can be observed that compared to conserved sequences without transposition, the S6PDH sequence in Amygdaloideae after transposition not only exhibits differences in protein activity but also demonstrates unique properties in transcript expression.

The rose also exhibited homeolog-expression bias, where homeolog from Clade 1 was more highly expressed than homeolog from the Clade 2. The expression trend of S6PDH proximal repeats mRNA was basically consistent with its enzyme activity. Exon deletion and length variation may be the key factors leading to the transcriptional expression of Clade2 sequence (*RochChr6G00021960*) and the deletion of protease activity. Although there was only one S6PDH sequence identified in strawberry, it exhibited notable expression levels in both leaves and young fruits. This finding is consistent with the observations made by S. Duangsrisai et al., who reported the detection of S6PDH mRNA expression in strawberry leaves and fruits (Duangsrisai et al., 2007).

### The evolutionary model of S6PDHs in Rosaceae

**Figure 7:**
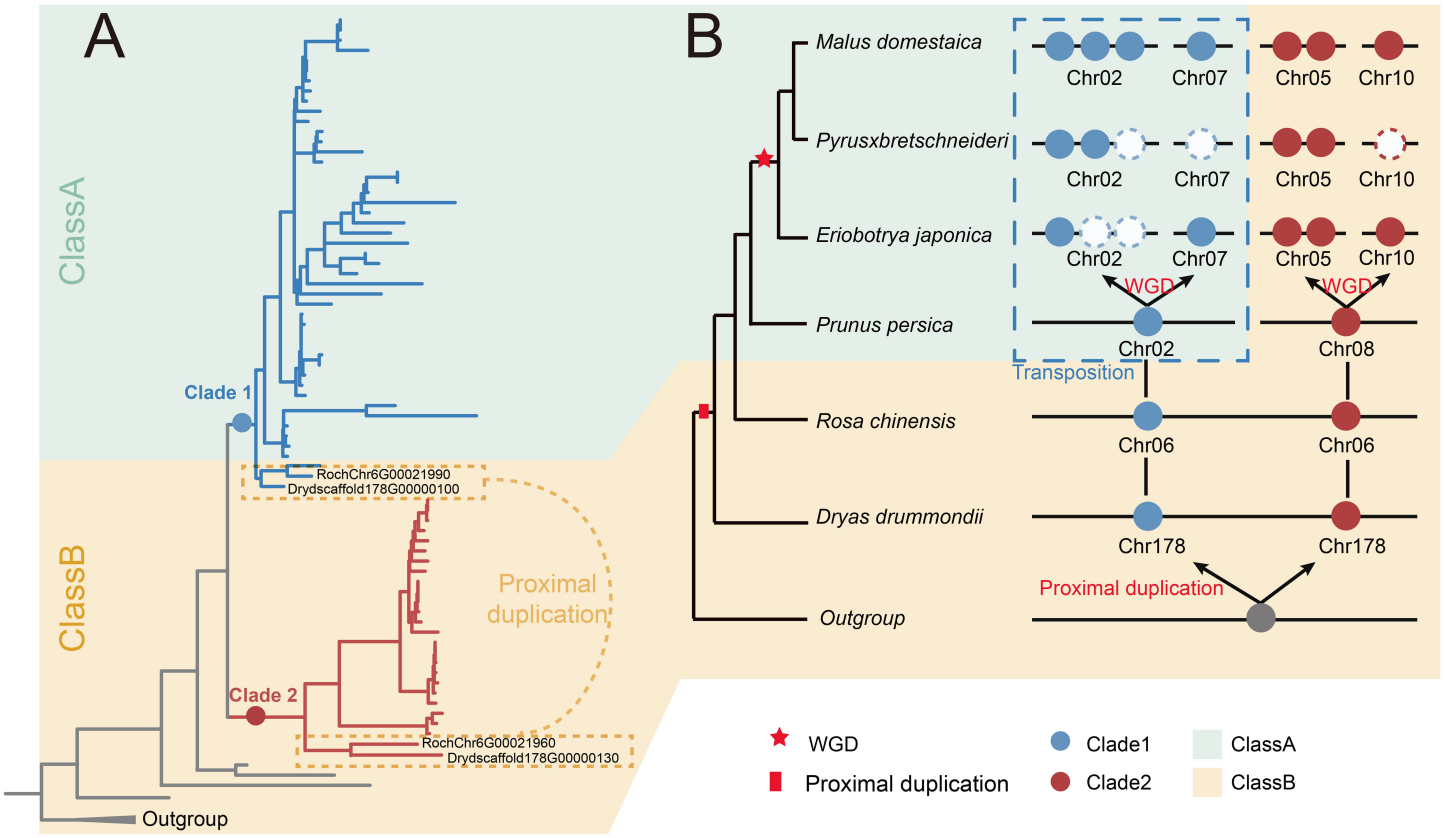
The evolution scenario of the S6PDH in Rosaceae. **(A)** The phylogenetic tree of S6PDH. The branches were colored blue or red based on their corresponding clade, Clade 1 and Clade 2, respectively. The background was colored in green and orange to represent the corresponding communities (Class A and Class B). The proximal gene pairs were highlighted. **(B)** The dynamic acquisition, loss, and transposition of S6PDH genes within Rosaceae. The phylogenetic tree of species is shown on the left side. The S6PDH gene distribution patterns are shown on the right. Genes within the same clades are represented by dots of matching colors, and genes within the same cluster share a background color. The colors were colored according to the Figure A.

In order to better understand the evolution of S6PDH gene, we constructed an evolutionary model of the Rosaceae S6PDH gene family, focusing on how the ancestral loci of Rosaceae gradually formed the present complex S6PDH gene lineage through extensive duplication and differentiation (**Figure 7**). The existing multi-copy pattern of Rosaceae S6PDH can be traced to a single proximal duplication event in the ancestor of Rosaceae. The duplication event resulted in the emergence of Clade 1 and Clade 2 ancestral genes. Subsequently, embedded within the intricate saga of chromosomal fission and fusion that unfolded in the evolutionary backdrop of the Amygdaloideae ancestor, a pivotal transposition event ensued. This genomic reshuffling involved one of the proximal duplicated S6PDH genes, culminating in its relocation and the consequent distribution of the S6PDH genes across two separate chromosomes within the Amygdaloideae lineage. The transposition was not merely a topological shift, it was concomitant with the emergence of novel regulatory expression, which were not only reflected in the decreased expression level and enzyme activity in leaves, but also presented a new regulatory mechanism of specific and sustained high expression at specific developmental stages or under specific environmental conditions. Such traits confer adaptive advantages in select ecological contexts. A recent WGD event occurred prior to the diversification of extant Malea (~ 35 - 54 MYA) contributed to the amplification in S6PDH copy numbers and paved the way for its eventual distribution onto four distinct chromosomes (Zhang et al., 2022, Zhang et al., 2023).

## Discussion

Sorbitol is recognized as a critical carbohydrate that plays a significant role in growth, development, and resistance of Rosaceae (Aprea et al., 2017). S6PDH is a key enzyme in sorbitol synthesis (Pleyerová et al., 2022). Understanding the evolutionary history of S6PDH not only sheds light on the metabolic pathways of sorbitol synthesis but also underscores the importance of gene duplication in the adaptation and resilience of Rosaceae species. By phylogenetic and synteny analysis, we have revealed that the origin of S6PDH in Rosaceae can be traced back to an ancestral proximal duplication event. This proximal duplication likely provided the genetic basis for the diversification of S6PDH within the family. Furthermore, the subsequent expansion of S6PDH genes in the Maleae tribe have been primarily driven by WGD events within this lineage (**Figure 2** and **Supplementary Figure 6**). Such genomic events are known to contribute to functional redundancy and the evolution of new traits, allowing for greater metabolic flexibility and adaptation to varying environmental conditions.

The cross clade proximal duplication of S6PDH was observed in Rosoideae and Dryadoideae. However, the S6PDH of Prunus tribe species deviated from the proximal arrangement pattern observed in Rosoideae and Dryadoideae species, instead being located on two distinct chromosomes (**Figures 2C, D**). The occurrence of this phenomenon is most likely due to multiple fission and fusion events during the evolutionary process of the ancestor genome of Plum, resulting in the subsequent separation and redistribution of the original adjacent genes onto distinct chromosomes (Zhang et al., 2012). The Maleae species demonstrated a higher degree of S6PDH multiplicity and possessed a greater number of copies of the S6PDH gene, which were distributed across four distinct chromosomes. For instance, apple and loquat S6PDH were located on chromosomes 2, 7, 5, and 10. Chromosome 2 and 7 were derived from a single primitive chromosome in the Maleae ancestor genome through a complex series of translocation and rearrangement events (Velasco et al., 2010; Zhang et al., 2022). Similarly, chromosomes 5 and 10 can also be traced back to a common ancestral chromosome (Velasco et al., 2010; Zhang et al., 2022). The fission/fusion event during the evolutionary process of the ancestral genome of Maleae could potentially account for the distribution of S6PDH genes on two distinct chromosomes, mirroring that observed in Prunus tribe species. Then the most recent whole-genome duplication (WGD) shared by Maleae (~ 35-54 Mya) allowed for the amplification of the number of S6PDH and ultimately resulted in its localization on four different chromosomes (Zhang et al., 2022, Zhang et al., 2023). S6PDH was distributed on four chromosomes in apples. Compared to the cultivated apple (cv. Golden Delicious, cv. Hanfu, and cv. Gala), wild apples (*Malus sieversii* and *Malus sylvestris*) contained a greater number of S6PDH copies (**Supplementary Table 6** and **Supplementarysaigure 2**). In comparison to wild species, the S6PDH gene located on chromosomes 5 and 10 of cultivated species neither contracted nor expanded. The difference in gene number between wild apple and cultivated apple was mainly observed in the sequence of chromosome 2. The ecological challenges confronting wild apple species are notably more intricate and dynamic. Given that sorbitol has been substantiated to confer substantial stress-resilient attributes, S6PDH, a pivotal gene in the biosynthesis of sorbitol, the preservation of a higher copy number might bestow upon wild apples enhanced metabolic flexibility and ecological adaptability. By contrast, the long-term domestication and artificial selection of cultivars to adapt to specific agricultural needs and environmental conditions may have led to a relative reduction in the number of copies of the S6PDH gene. The fungal pathogen *Alternaria alternata* is known to disrupt plant metabolism, causing black necrotic leaf spots and potentially leading to leaf necrosis (Zhang et al., 2018). Transcriptome analysis revealed a marked upregulation of S6PDH on chromosome 2 upon infection of apple leaves by *Alternaria alternata*, indicating that S6PDH on chromosome 2 might have the ability to regulate metabolism to resist fungal diseases (**Figure 6** and **Supplementary Figure 7**). Wild apple species were generally considered to have a strong ability to resist fungal infection (Duan et al., 2017). The retention of more stress-specific S6PDH copies might be one of the defensive strategies developed through long-term natural selection in their natural habitats. In the breeding process of cultivated apple varieties, certain S6PDH copy sequences on chromosome 2 might be inadvertently eliminated due to linkage with genes expressing undesirable traits, thus contributing to a relative decrease in the overall S6PDH copy number (Sun et al., 2020).

The phylogenetic analysis revealed that S6PDH of Rosaceae can be categorized into two clades: Clade 1 and Clade 2, both encompassing species sequences from the three subfamilies of Rosaceae. By integrating synteny analysis, lineage-specific transposition was identified in peach subfamily sequences within Clade 1 (**Figure 2**). The comparison of the S6PDH gene on enzyme activity and leaf transcriptional regulation mode revealed that the functionality of the Clade 2 S6PDH sequence was superior. In contrast, the transcriptional expression level of the Amygdaloideae transposition S6PDH in Clade 1 was relatively low in the major functional regions (leaves), and the purified protein also exhibited decreased enzyme activity. According to the previous study, we thought that the transposition was the mainspring for the for the decreased S6PDH enzyme activity. Transposed genes may lose their upstream regulatory region, resulting in attenuation or silencing (Roberts and Morris, 2013). The follow-up research could investigate the upstream promoter regulatory region or protein conformation. Exon deletion and length variation might be the key factors leading to the loss of Clade 2 transcriptional expression and the reduction in protease activity for proximal duplicated gene pairs belonging to the same synteny cluster (Class B) in Rosoideae and Dryadoideae (**Figure 5**). It is noteworthy that, although the expression of the transposition gene (*Mgde07G1054400*) in Clade 1 was reduced in the leaves, it exhibited continuous and specific expression in the fruits postharvest. Furthermore, the functional characteristics of the transposition genes (*Mgde02G1264100*, *Mgde02G1263800*, and *Mgde02G1264600*) were enhanced under biological stress. By contrast, the expression levels of S6PDH sequences in Clade 2 were found to be higher during leaf and late stage of fruit development, as well as under abiotic stress conditions (**Figure 6**). The findings of studies have confirmed that translocation genes are more likely to differentiate in gene expression patterns (Cheng et al., 2013). Compared to their proximal duplicated counterparts, transposed genes were more prone to differentiation, displaying divergent expression (Feng et al., 2022; Massey et al., 2021). The duplicated genes in the Amygdaloideae of S6PDH indeed displayed distinct enzymatic phenotypes and showed more targeted functional differentiation in specific tissues along with transposition. It seems that the gene transposition with similar sequence but altered chromosome position information greatly improves the complexity of S6PDH gene. The multiple copies of S6PDH have evolved into a wide range of functional roles, which are more propitious to the adaptability of the Amygdaloideae.

As an “anomaly” in the Rosoideae, strawberries transport sucrose as the main carbohydrate to the fruit, in stark contrast to other members where sorbitol predominates (Duangsrisai et al., 2007). Research indicates that strawberries contain only trace amounts of sorbitol, markedly lower when juxtaposed against the Amygdaloideae (Duangsrisai et al., 2007; Ogiwara et al., 1998). Strawberries possess a single copy of S6PD, which exhibits high transcriptional expression and enzymatic activity. The S6PDH expression in strawberries surges during the inception of fruit development, eclipsing its presence in source leaves, suggesting an important role of S6PDH in the fruit (**Figure 3** and **Figure 6H**). Given the compensatory relationship between sorbitol and sucrose (Teo et al., 2006), we speculated that strawberries were mainly rely on sucrose, metabolism under environment, weakens the metabolism of sorbitol. sorbitol synthesis appears chiefly consigned to the maintenance of local metabolic homeostasis or the provision of localized, temporally confined safeguarding. Akin to Arabidopsis, a non-Rosaceae plant that was sucrose oriented and did not utilize sorbitol as the main transportable photosynthate. S6PDH in Arabidopsis was enriched and expressed specifically in silique rather than in source leaves, and the content of sorbitol increased only under specific stress conditions, which was almost undetectable in conventional growing environment (Rojas et al., 2019).

Furthermore, S6PDH genes have also been identified in several non-Rosaceae species, including *Glycine max*, *Vitis vinifera*, *Oryza sativa*, *Brachypodium distachyon*, *Sorghum bicolor*, and *Zea mays* based on homology (Yadav and Prasad, 2014; Almaghamsi et al., 2021; Hartman et al., 2017). Similar to Arabidopsis, the number of S6PDH genes in these species is limited, with most possessing only a single copy. In these organisms, sorbitol is upregulated only in specific tissues or under specific stresses. It is noteworthy that a protein study of S6PDH in rice revealed that the Michaelis-Menten constant (Km) values for rice S6PDH with G6P and S6P were 15.9 ± 0.2 and 7.21 ± 0.5 mM, respectively. This indicates that rice S6PDH has a higher affinity for S6P and a greater catalytic efficiency for the conversion of S6P to G6P (Yadav and Prasad, 2014). In contrast, S6PDH in Rosaceae species exhibits a more prominent catalytic conversion of G6P to S6P. The limited availability of the S6P substrate, which is necessary for sorbitol synthesis, may explain why non-Rosaceae species, such as rice, exhibit very low or undetectable levels of sorbitol in conventional environments, despite the presence of S6PDH. This suggests that the regulatory mechanisms governing sorbitol metabolism may differ between Rosaceae and non-Rosaceae species, emphasizing the importance of ecological context and gene regulation in the metabolic pathways of different plant families.

The study utilized a total of 16 genomes from the Rosaceae lineage, encompassing species belonging to Amygdaloideae (n=13), Rosoideae (n=2), and Dryadoideae (n=1). Due to the limited availability of genomic data for Dryadoideae, only one genome was accessible for this subfamily. The disparity in the number of species may impose certain constraints. However, our findings still offer significant preliminary insights. The inclusion of additional genomic data, particularly from Rosoideae and Dryadoideae, in future studies might further bolster the reliability of our conclusions and facilitate a more comprehensive understanding of the S6PDH gene family.

## Data Availability

The datasets presented in this study can be found in online repositories. The names of the repository/repositories and accession number(s) can be found in the article/**Supplementary Material**.
