# Supplementary material for: Evolutionary dynamics and functional characterization of proximal duplicated sorbitol-6-phosphate dehydrogenase genes in Rosaceae
Source: Front Plant Sci. 2024 Nov 8;15:1480519. doi: 10.3389/fpls.2024.1480519 (PMC11581945; doi:10.3389/fpls.2024.1480519)
Supplement: Supplementary file 1 [file DataSheet1.docx]

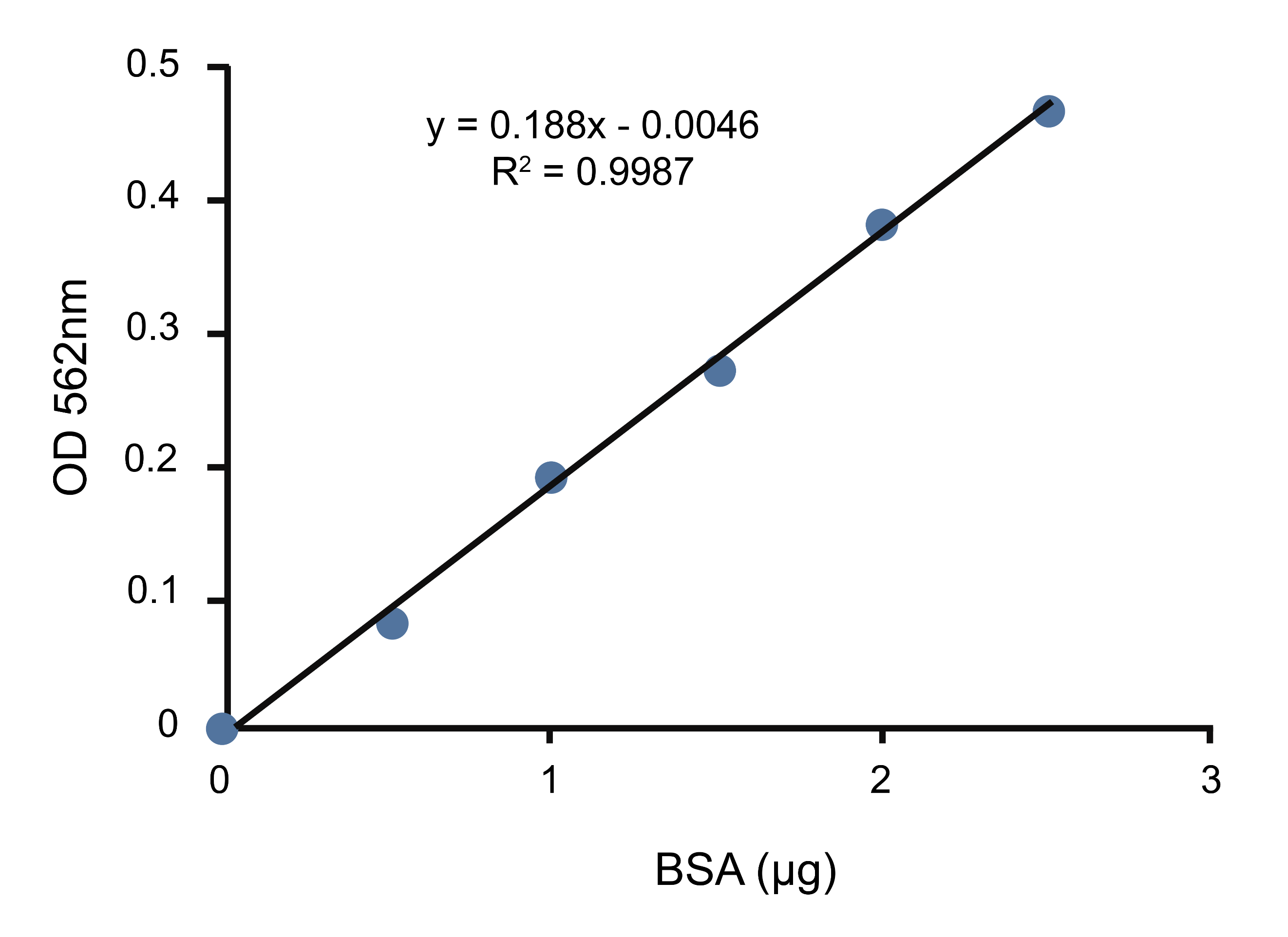
 **Supplemental Figure 1. BCA standard curve drawing.**

**
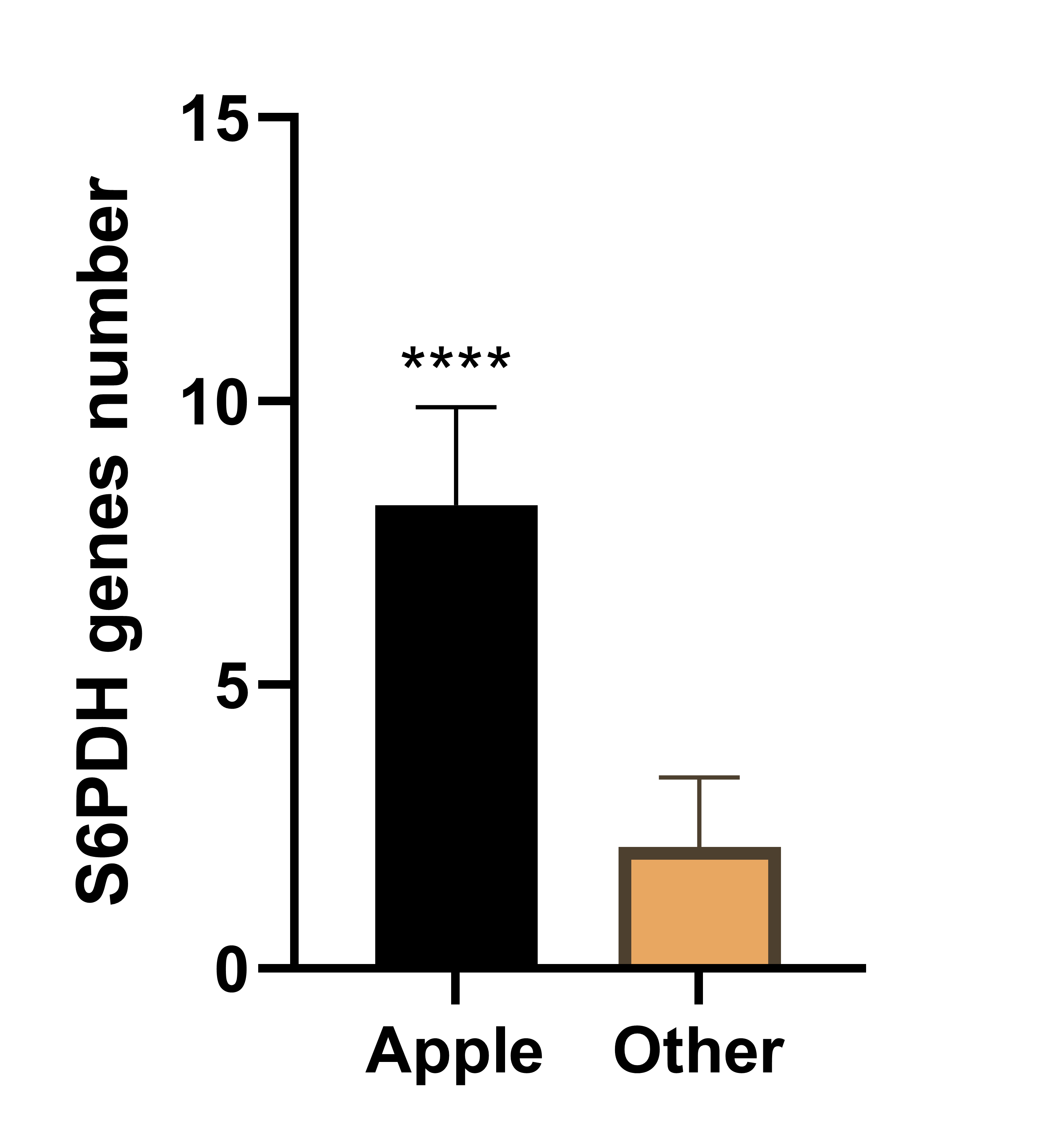
**

**Supplemental Figure 2. Number of S6PDH genes.** ** indicates significant difference at the 0.01 levels by *t*-test.


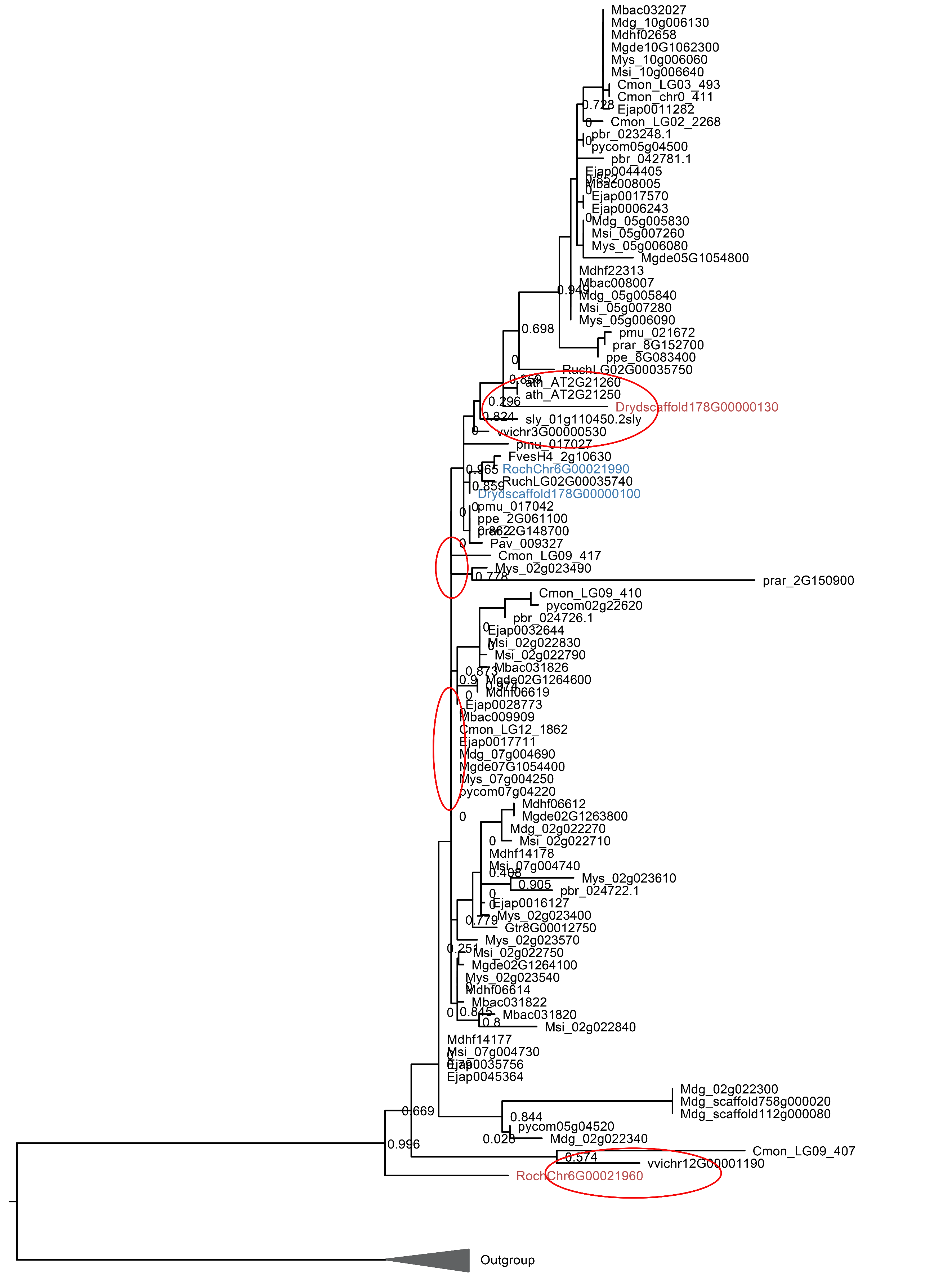


**Supplemental Figure 3. An example of the phylogenetic tree constructed based on protein sequences exhibited a topological structure that was unable to accurately reflect the relationships between species.**


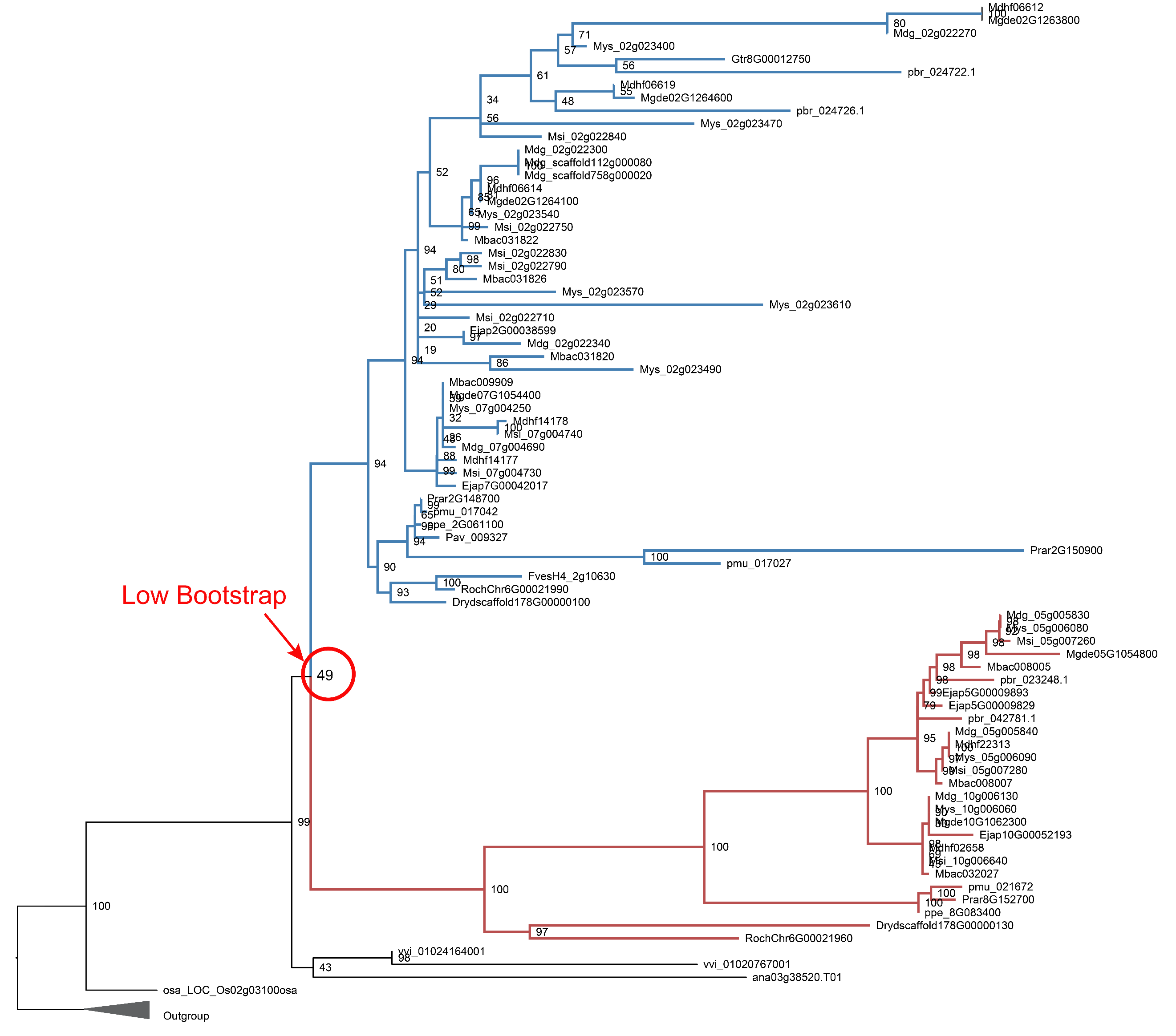


**Supplemental Figure 4. An example of a phylogenetic tree reconstructed from protein sequences showed weak bootstrap support.**

**
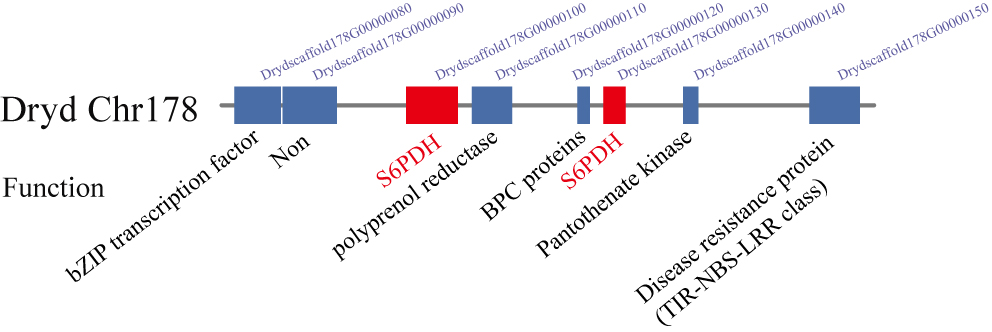
**

**Supplemental Figure 5. Functions of the genes located upstream and downstream of S6PDH in *Dryas drummondii*.**

**
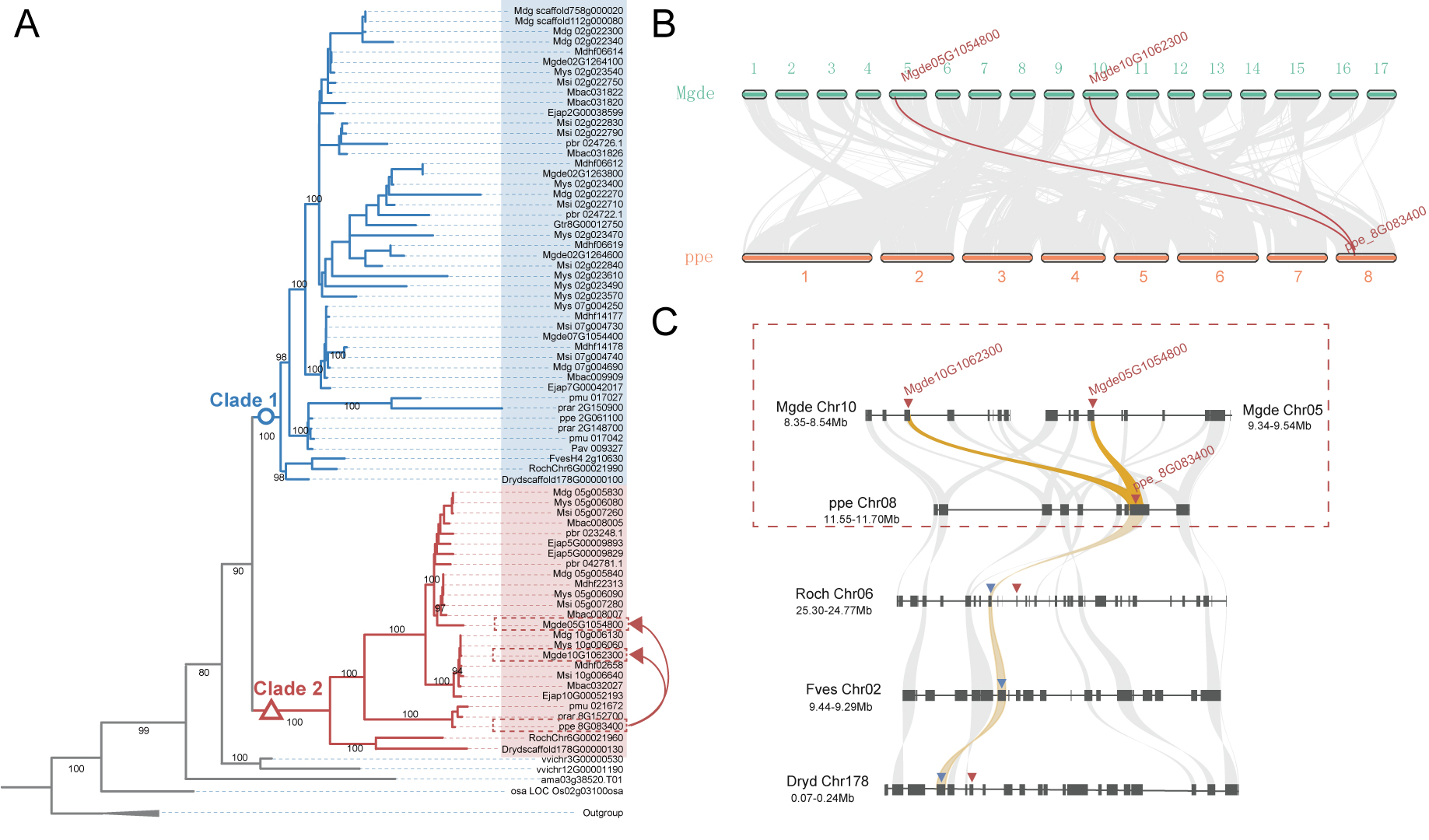
**

**Supplemental Figure 6. Phylogenetic and synteny relationships of S6PDH in Clade 2 of *Prunus persica* and *Malus domestica*.**


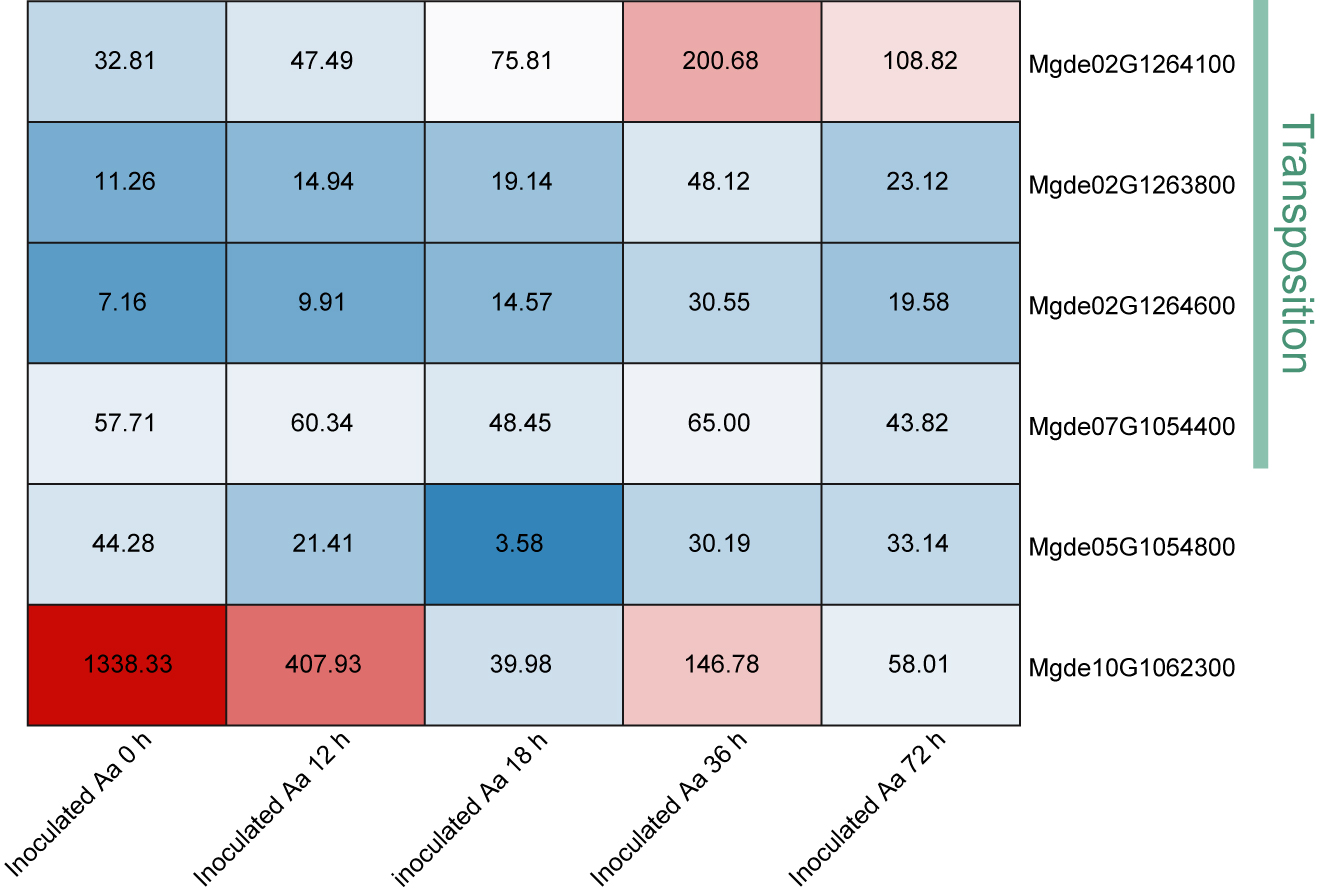


**Supplemental Figure 7. Expression patterns of S6PDH in apple under biotic stresses.**
